# RETRACTION: Antihyperglycemic, Antidiabetic, and Antioxidant Effects of *Garcinia pedunculata* in Rats

**DOI:** 10.1155/ecam/9807175

**Published:** 2026-07-28

**Authors:** Evidence-Based Complementary and Alternative Medicine

RETRACTION: M. Y. Ali, S. Paul, E. M. Tanvir, et al., “Antihyperglycemic, Antidiabetic, and Antioxidant Effects of *Garcinia pedunculata* in Rats,” *Evidence-Based Complementary and Alternative Medicine*, 2017, 2979760, https://doi.org/10.1155/2017/2979760.

The above article, published online on 19 October 2017 in Wiley Online Library (https://wileyonlinelibrary.com), has been retracted by John Wiley & Sons Ltd. The retraction has been agreed due to concerns identified in the figures, some of which have been raised on PubPeer [1]. A summary of the concerns is below:

• In Figure 11, panel C shares unexpected repeated areas with:
  ◦ The Gp‐heart panel in Figure 6 of [2], which shares some of the same authors
  ◦ The pomelo‐heart panel in Figure 6 of [3], which shares some of the same authors
• In Figure 12, there are unexpected, repeated areas between panels C and F when rotated 270°

After assessment of the concerns and the author’s response, the results and conclusions can no longer be considered reliable, and the article is, therefore, retracted.

Yousuf Ali disagrees with the retraction, whilst no response was received from the remaining authors.
